# Case Report: Awake lateral decubitus intubation for a patient with critical tracheal stenosis secondary to retrosternal goiter: salvaging a 2 mm airway without ECMO support

**DOI:** 10.3389/fonc.2026.1712391

**Published:** 2026-02-16

**Authors:** ShuiJing Wu, BingDuo Wang, Ping Cui, Hui Ye, ShengWen Song, XiangMing Fang, GuoHao Xie

**Affiliations:** 1 Department of Anesthesiology, The First Affiliated Hospital, School of Medicine, Zhejiang University, Hangzhou, Zhejiang, China; 2 Zhejiang University School of Medicine, Hangzhou, Zhejiang, China

**Keywords:** a dual-guidance technique, difficult airway, lateral decubitus, retrosternal goiter, tracheal stenosis

## Abstract

**Background:**

Tracheal compression frequently occurs in patients with retrosternal goiter, potentially leading to critical tracheal stenosis. The lateral position has been shown to increase the cross-sectional area of the upper airway and improve the oxygenation in sedated patients compared to the supine position. However, it is unclear whether it can be successfully applied to airway management in patients with critical tracheal stenosis.

**Case presentation:**

We report the case of a 71-year-old female patient undergoing total thyroidectomy for a massive retrosternal goiter extending to the descending aorta. Chest computed tomography (CT) showed a tracheal lumen with a minimum diameter of 2.0 mm when the patient was in the supine position. Consequently, our anesthesiology team adopted a 30° head-up left lateral positioning strategy, integrating video laryngoscopy and fiberoptic bronchoscopy (FOB) for awake intubation as the primary airway management protocol. Extracorporeal membrane oxygenation (ECMO) was prepared as a backup for airway rescue. Ultimately, the patient successfully established an artificial airway via awake intubation in the lateral decubitus position, followed by the smooth completion of a thyroidectomy.

**Conclusion:**

Employing the lateral decubitus position, a dual-guidance technique combining video laryngoscopy with FOB for awake endotracheal intubation, represents a feasible airway management strategy for patients presenting with critical tracheal stenosis.

## Introduction

The incidence of retrosternal goiter ranges from 0.02% to 15%, with its characteristic pathological feature being the downward extension of cervical thyroid tissue along the cervicomediastinal fascial space (1). As the disease progresses, the swelling can compress the trachea, leading to mechanical stenosis (2). Data from tertiary hospitals indicate that tracheal compression results in upper airway obstruction in 14–31% of goiter cases, with retrosternal goiters exhibiting compression in 35–73% of thyroidectomy patients (3). Among these cases, 1–2% may progress to critical airway stenosis (4).

Notably, a 1% decrease in airway diameter corresponds to a reduction in peak expiratory flow by 0.03 L/min, thereby directly compromising respiratory function (5). Severe subglottic stenosis characterized by an airway diameter under 5 mm is associated with a significantly elevated risk of ‘cannot intubate, cannot oxygenate’ (CICO) events (6, 7), a life-threatening emergency with nearly 50% mortality (8). A systematic review indicated that 62% of previous cases of extremely severe stenosis necessitated the use of ECMO support (9). Nevertheless, ECMO was associated with a 30-day mortality rate of 54%, with major complications including renal failure requiring continuous venovenous hemofiltration (52%), bacterial pneumonia (33%), and sepsis (26%) (10). Previous studies suggest that postural interventions could provide a more effective solution (11). A pediatric study demonstrates that adopting a lateral position can increase the tracheal cross-sectional area by 40–50% relative to the supine position (12). However, the efficacy of this method in adult patients with critically severe stenosis has yet to be established.

Here, we report an interesting case of a 71-year-old female patient with critical tracheal stenosis secondary to a retrosternal goiter. A prominent triple retraction sign was observed even at rest, strongly suggesting a critical airway obstruction. Given the progressively worsening dyspnea and the imminent risk of suffocation, an urgent retrosternal thyroidectomy was performed to relieve tracheal compression. An awake endotracheal intubation in the lateral decubitus position was successfully executed to establish a secure airway. This literature delineates the challenges associated with managing a difficult airway during retrosternal thyroidectomy in the presented patient and offers a comprehensive review of perioperative strategies for managing airway obstruction caused by retrosternal goiter.

## Case presentation

### Patient information

The patient was a 71-year-old female, with a 6-month history of progressive dyspnea, which worsened over the past week with new-onset neck compression and hoarseness. She had a known history of thyroid nodules for over 20 years, with notable enlargement observed over the past five years. Her clinical manifestations included persistent and intensifying dyspnea, pronounced anterior neck pressure, and vocal impairment, all of which substantially compromised her daily functioning. Additionally, she reported orthopnea during daytime activities and frequent nocturnal awakenings due to dyspnea, with only transient symptomatic relief achieved in the left lateral decubitus position. Her past medical history was significant for hypertension and bronchiectasis.

The physical examination revealed a body mass index of 19.78 (159 cm, 50 kg) and significant asymmetric neck thickening, particularly on the left side. Palpation of the left neck revealed a firm, non-tender multinodular mass with slight mobility during swallowing. Rightward tracheal deviation and a scabbard-like deformity palpable superior to the suprasternal notch were also observed. At baseline, the patient exhibited a pulse oximetry saturation (SpO2) of 85% on room air (FiO_2_ 0.21), which increased to 93% following administration of supplemental oxygen via nasal cannula at 3 L/min (estimated FiO_2_ 0.30). A prominent triple retraction sign (intercostal, suprasternal, and subcostal) and inspiratory stridor were observed in the patient. Auscultation demonstrated a fixed laryngeal wheeze, consistent with a fixed upper airway obstruction. Neurologic examination identified mild hoarseness without complete aphonia, alongside an intact cough reflex, suggesting possible mild compression of the recurrent laryngeal nerve but not total nerve injury.

**Figure 1:**
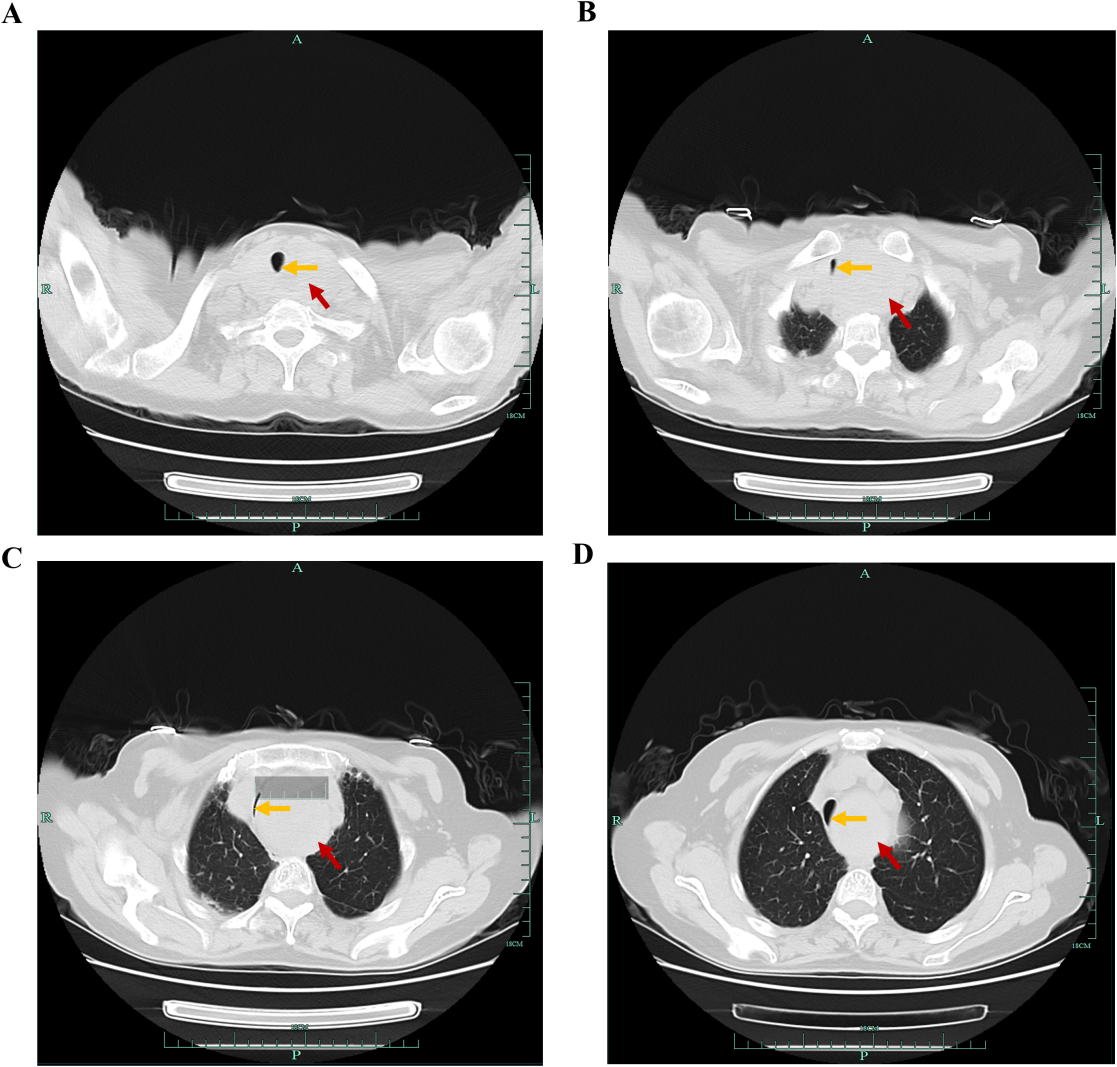
Preoperative computed tomography (CT) imaging of the head and neck. The CT scan of the neck and chest showing a large mass situated posterior to the left side of the sternum, causing substantial tracheal stenosis **(A-D)**. The narrowest tracheal diameter in the supine position is approximately 2.0 mm **(C)**. Red arrow is the mass; Yellow arrow is the narrowed tracheal compressed by the mass.

**Figure 2:**
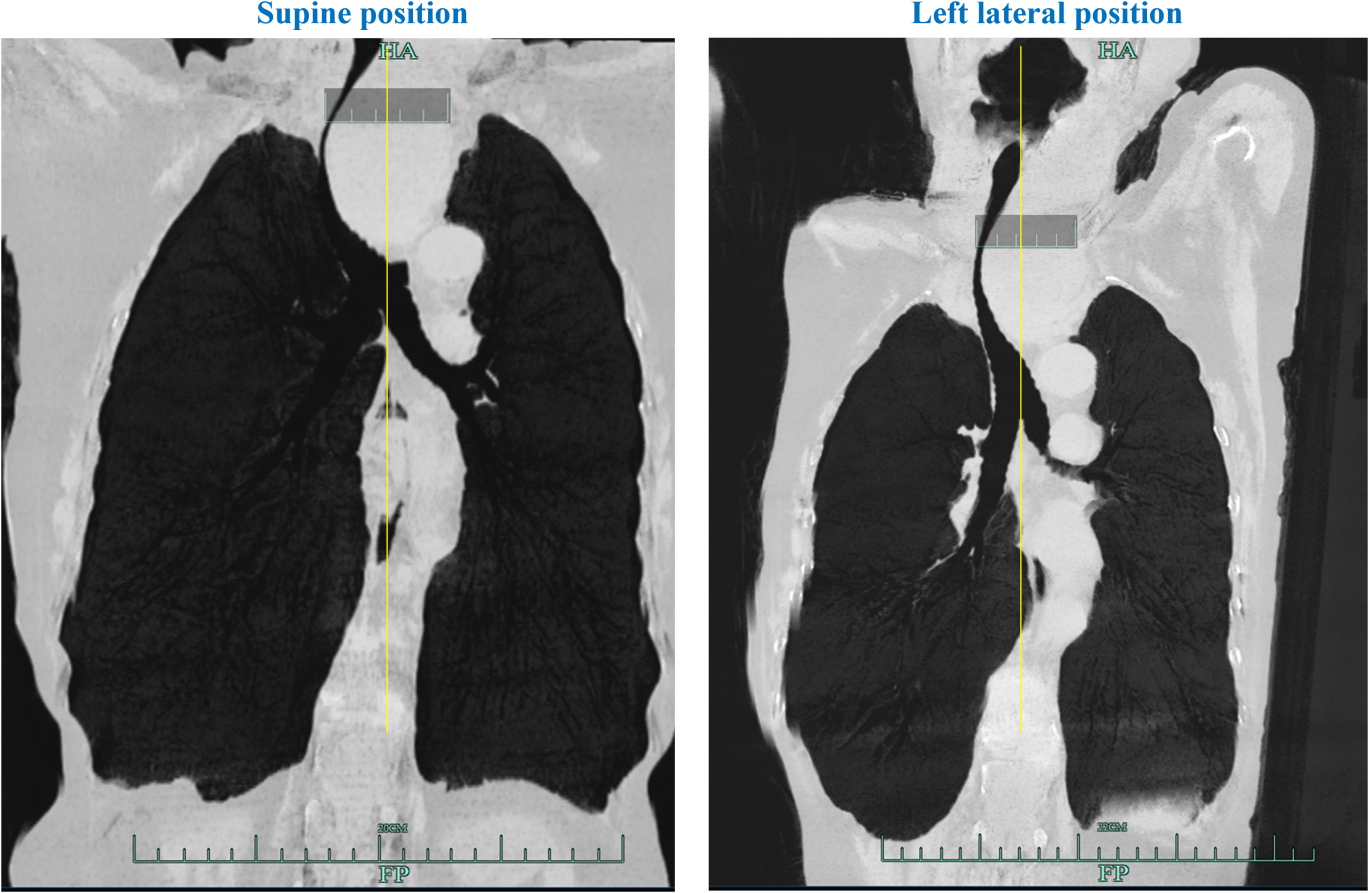
The imaging of coronal computed tomography (CT). The coronal CT scan of the airway showing that left lateral decubitus positioning results in an attenuated rightward tracheal deviation and a significant increase in the tracheal lumen diameter at its narrowest point.

Bilateral thyroid ultrasonography identified multiple nodules. On the left side, two larger nodules measuring approximately 4.3 × 2.6 cm and 5.6 × 4.8 cm were observed, each resulting from the confluence of multiple nodules. On the right side, a larger nodule measuring approximately 3.7 × 3.3 cm was identified, characterized as a cystic-solid mass containing multiple hyperechoic foci, also resulting from the fusion of multiple nodules (**Supplementary Figure S1**). A computed tomography (CT) scan of the neck and chest revealed a 5 × 8 cm mass situated posterior to the left side of the sternum. This mass caused substantial tracheal stenosis, with the narrowest tracheal diameter measuring 2.0 mm in the supine position (**Figure 1**). Additionally, lung CT imaging demonstrated bronchial dilation bilaterally, accompanied by minimal peribronchial inflammation and the presence of mucus plugs within certain cavities. Following a multidisciplinary team consultation, a left lateral airway reconstruction was undertaken to assess the effect of left lateral positioning on the extent of airway stenosis. This intervention was prompted by the patient’s reported improvement in dyspnea when positioned in the left lateral decubitus posture, which corresponded with an increase in the tracheal lumen diameter to 3.5 mm at its narrowest site (**Figure 2**).

### Endotracheal intubation procedure

The anesthesia team developed an awake intubation plan with ECMO as a backup. Following ultrasound guidance, the left radial artery was catheterized, and single-lumen arrow catheters were introduced into the right femoral vein and the internal carotid vein. The patient was then placed in a modified left lateral decubitus position with the head elevated approximately 30° (an optimization of the sniffing position). Pre-oxygenation with high-flow nasal cannulae was used during the preoperative preparation phase, raising the blood oxygen saturation to over 98%. In order to reduce the patient’s tension and anxiety, and to lower the airway reactivity during intubation, remifentanil was infused intravenously at a rate of 1.5 μg/kg/h. Simultaneously, 2% lidocaine nebulization inhalation (lasting 10 minutes) was implemented to ensure comprehensive anesthesia of the airway mucosa, thereby minimizing the risk of coughing and laryngeal spasm associated with laryngoscopy and intubation. In addition, to prevent mucosal edema and mitigate injury from repeated intubation attempts in cases of difficult airway management, an intravenous injection of 40 mg methylprednisolone was administered. Prior to intubation, a fiberoptic bronchoscope (FOB) was inserted nasally for airway assessment, which confirmed normal tracheal anatomy with a patent lumen. Additionally, 10 mL of 2% lidocaine was administered through the bronchoscope’s working channel to reinforce topical anesthesia of the glottic region.

**Figure 3:**
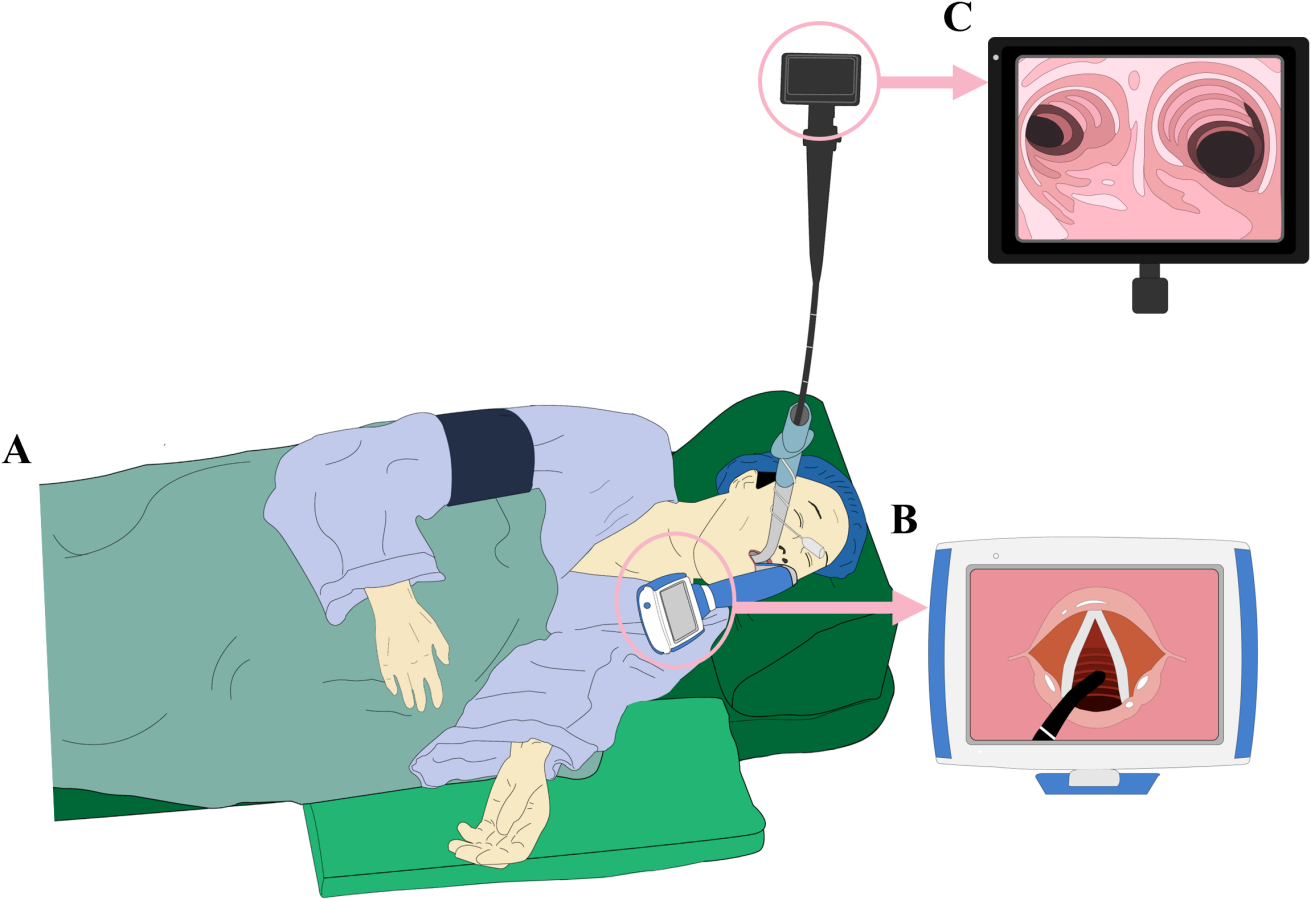
A dual-guidance technique combining video laryngoscopy with fiberoptic bronchoscope (FOB). The patient was placed in a modified left lateral decubitus position with the head elevated approximately 30° **(A)**. Initially, the video laryngoscope was employed to expose the glottis **(B)**. Subsequently, a 4.8 mm outer diameter FOB equipped with a suction channel was orally introduced to assist in directing a 6.5 mm reinforced endotracheal tube (ETT) toward the glottis under continuous direct visualization **(B, C)**. Finally, The FOB then advanced smoothly through the glottis, allowing for accurate placement of the ETT approximately 2 cm above the carina **(C)**.

Following comprehensive topical airway anesthesia, a dual-guidance technique combining video laryngoscopy with FOB was implemented under the direct supervision of a senior attending physician (**Figure 3**), which has been utilized in patients with predicted difficult airways and improved intubation outcomes (13). The detailed procedure was conducted collaboratively by two experienced attending physicians. Initially, the video laryngoscope was employed to expose the glottis, achieving a Cormack-Lehane grade II view. Subsequently, a 4.8 mm outer diameter FOB equipped with a suction channel was advanced orally to facilitate the guidance of a 6.5 mm reinforced endotracheal tube (ETT) toward the glottis under continuous direct visualization. The FOB then smoothly traversed the glottis, enabling precise ETT placement in the mid-trachea. Final positioning of the ETT was confirmed with the tube tip positioned 24 cm from the incisors (direct visualization via bronchoscope confirmed placement approximately 2 cm above the carina). Following tube fixation and connection to the ventilator, three consecutive respiratory cycles demonstrated stable end-tidal carbon dioxide waveforms (P_ET_CO_2_ 35-45mmHg), with symmetrical bilateral breath sounds confirming proper tube positioning. The entire procedure was completed within 15 minutes, during which the patient maintained stable hemodynamic parameters (blood pressure fluctuations <20% of baseline) without episodes of hypoxemia (SpO_2_ consistently ≥92%) or other complications.

### Anesthesia maintenance and surgical procedure

**Figure 4:**
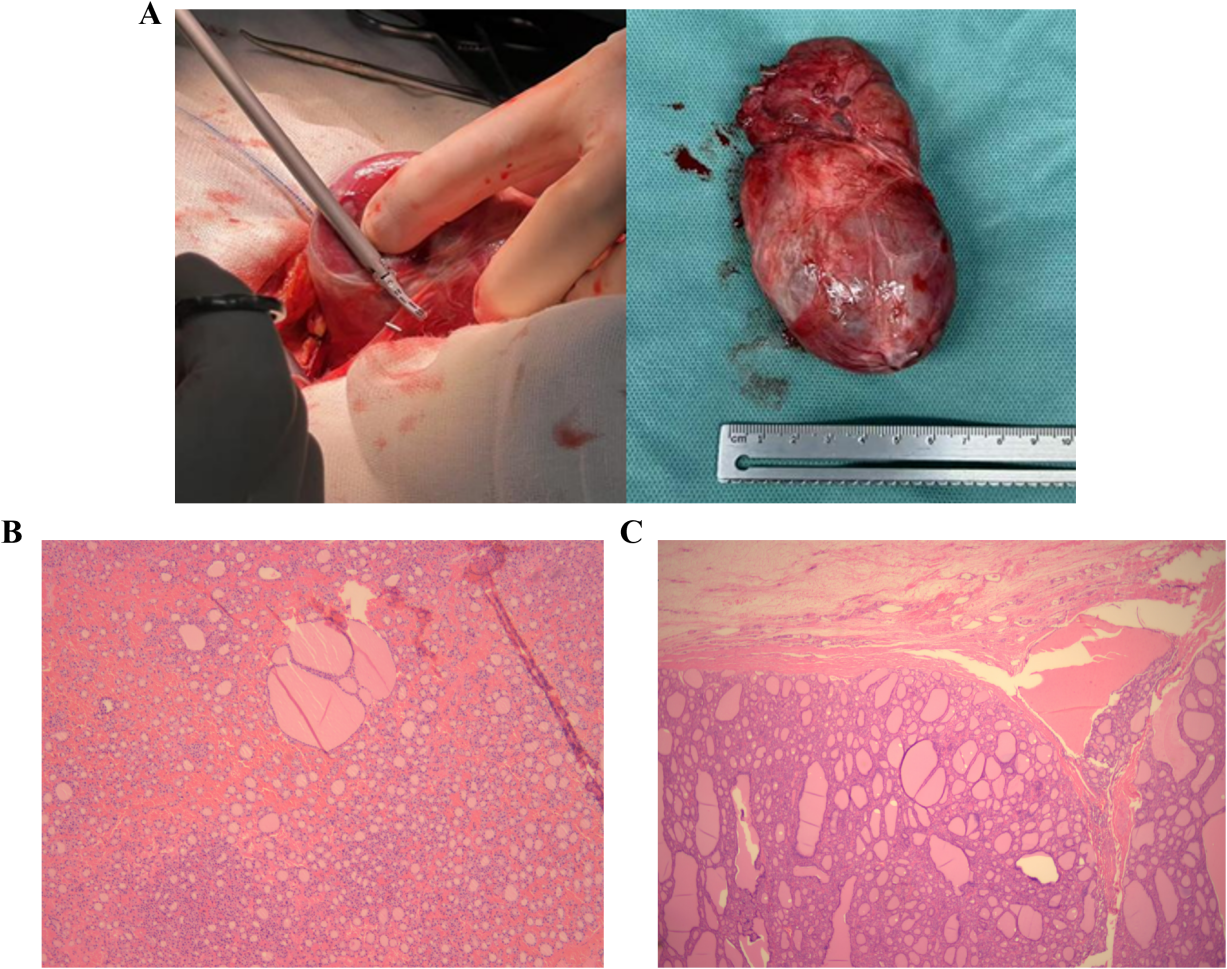
Resected retrosternal thyroid goiter and postoperative pathology. The thyroid mass was entirely excised, measuring approximately 7 × 10 centimeters **(A)**. Postoperative pathology of the retrosternal thyroid mass **(B, C)**.

Following successful establishment of an artificial airway via awake endotracheal intubation in the lateral decubitus position, etomidate (15 mg), sufentanil (25 µg) and vecuronium (8 mg) were immediately administered intravenously. General anesthesia was then maintained with a continuous infusion of propofol and remifentanil, combined with 1% sevoflurane inhalation. Subsequently, the patient was positioned supine in preparation for the surgical procedure. Finally, the patient successfully underwent a left posterior thoracic thyroidectomy, total thyroidectomy, and parathyroid autotransplantation during general anesthesia. The thyroid mass situated posterior to the sternum was entirely excised, measuring approximately 7 × 10 centimeters (**Figure 4A**).

### Postoperative management and outcomes

The patient was transferred to the intensive care unit (ICU) for close monitoring after surgery, where his vital signs remained stable and his respiratory function recovered well. Twenty-four hours after surgery, respiratory training (pressure support ventilation 8 cmH_2_O + positive end-expiratory pressure 5 cmH_2_O, inspired oxygen fraction (FiO_2_) 30% for 60 minutes) demonstrated good respiratory function: respiratory rate 11 breaths/min, tidal volume 388 mL. Arterial blood gas analysis revealed pH 7.47, PaCO_2_ 34.0 mmHg, PaO_2_ 178.0 mmHg, hemoglobin 12.4 g/dL, HCO_3_^-^ 24.7 mmol/L, and actual base excess +1.4 mmol/L, indicating stable respiratory function. The ETT was successfully removed on postoperative day 1 (POD1). To prevent airway collapse following extubation, we initially placed an exchange catheter to facilitate potential re-intubation. After tracheal tube removal, and the patient maintained stable breathing, oxygenation, and vital signs, the exchange catheter was withdrawn. The patient was then transferred to the general ward on POD2 for continued specialized care.

Postoperative pathology revealed a left retrosternal thyroid follicular adenoma (6.5 × 5.0 × 2.5 cm) with a nodular goiter, and a right thyroid follicular tumor of uncertain malignant potential (3.0 × 2.0 × 1.5 cm) with a nodular goiter (**Figures 4B, C**). Molecular testing confirmed BRAF wild-type status, and immunohistochemistry revealed CD56 (+), CK19 (+), and BRAF-V600E (-). Postoperative reevaluation using lung CT (**Supplementary Figure S2**) and chest radiography (**Supplementary Figure S3**) showed that the patient’s tracheal was centered, with complete resolution of the airway stenosis. Ultimately, the patient was successfully discharged from the hospital on POD10.

## Discussion

Airway management is generally smooth in most patients presenting with goiter, but patients with giant retrosternal goiters may face severe challenges (1). Careful airway evaluation is imperative in these cases. Although traditional assessment tools, such as the Mallampati classification, can predict challenges related to laryngoscopic visualization, they are insufficient for determining the likelihood of endotracheal tube passage through areas of tracheal compression (14). Therefore, imaging modalities, particularly CT, are essential to precisely identify the location and extent of airway obstruction (15). During induction of anesthesia, administration of muscle relaxants may exacerbate partial airway obstruction, potentially progressing to complete obstruction (16). Additionally, physiological alterations including cephalad displacement of the diaphragm, can worsen ventilatory impairment and increase the risk of mask ventilation failure (17). In the present case, the patient exhibited dyspnea at rest, and CT imaging confirmed severe tracheal compression. Therefore, the experts unanimously recommended awake fiberoptic bronchoscopic intubation in the left lateral decubitus position (the patient is lying on the left side, so the left side is down and the right side is up) as the preferred option (18). This technique preserves spontaneous ventilation while reducing the risks of aspiration and airway collapse. Moreover, preparations for advanced life support measures, such as ECMO, were deemed critical. A multidisciplinary team comprising anesthesiology and thoracic surgery specialists was convened, and a detailed emergency response plan was established to facilitate prompt intervention in the event of acute complete airway obstruction.

To establish an artificial airway for this patient, we implemented an optimized intubation position with the patient in a 30° left lateral decubitus position. This intervention is expected to alleviate the tumor-induced compression on the airway, decrease the rightward deviation of the trachea, and enlarge the lumen diameter at the narrowest segment of the patient’s trachea. These effects were first corroborated by chest coronal CT imaging of the airway obtained in the left lateral decubitus position in this study. Based on anatomical considerations for such patients and the updated American Society of Anesthesiologists Practice Guidelines for Management of the Difficult Airway (19), our optimized airway management protocol included: 1) Multidisciplinary team-based preoperative simulation training; 2) Patient positioning in the left lateral decubitus posture with a head-up tilt, accompanied by carefully titrated sedation to preserve spontaneous ventilation; 3) Preparation for ECMO standby, and a single-lumen Arrow catheter pre-inserted into the right internal jugular and femoral veins to facilitate rapid ECMO initiation in the event of an emergent airway complication during intubation; 4) Administration of 2% lidocaine for totally topical mucosal anesthesia; 5) The implementation of a dual-guidance technique combining real-time video laryngoscopy with FOB aims to enhance safety of intubation and mitigate the potential airway injury. In previous difficult airway cases, this protocol was also successfully applied for awake tracheal intubation, with all procedures completed within 15 minutes, significantly faster than conventional ECMO-assisted intubation, which typically requires a minimum of 20 minutes for preparation (20). Health economic evaluations showed that compared with traditional ECMO-assisted approaches, this method saved an average of $2,518 to $200,658 per patient, primarily attributable to reductions in ECMO consumables and ICU hospitalization expenses (21). Furthermore, the incidence of severe complications including major bleeding, thrombosis, infection, and pneumothorax was reduced from a baseline rate of 50–70% to zero (22, 23). These findings underscore the considerable potential of this protocol for widespread implementation in primary healthcare settings.

The airway management of the present case offers several novel insights. Our exploratory evaluation of adult patients demonstrated that body positioning may affect mediastinal anatomy, prompting us to propose a tentative “ECMO backup-optional” clinical strategy to optimize the treatment of patients with severe tracheal stenosis while balancing safety and cost-effectiveness. The implementation of a dual-visualization approach combining video laryngoscopy with FOB, complemented by left lateral tilt with slight head elevation, appeared to improve glottic visualization success and potentially reducing intubation related trauma. This position allows gravity to naturally droop the tongue and significantly improves glottic exposure, as evidenced by an average improvement of one grade in the Cormack-Lehane classification (24). Additionally, lateral position has been shown promising benefits for surgical patients including improved thoracic compliance, reduced airway closing pressure, and possible prevention of intraoperative hypoxemia (25, 26). The critical consideration lies in selecting the appropriate lateral position, whether left or right. It can be informed by the patient’s clinical presentation, specifically whether adopting a particular lateral position alleviates respiratory distress. Additionally, the assessment should further incorporate the anatomical location of the mass demonstrated by neck CT imaging. The inherent mobility of thyroid masses allows them to shift under gravity, thereby alleviating airway compression. Therefore, the left lateral position should be adopted for a mass situated on the left posterior aspect of the trachea, and the right lateral position for one on the right. In the present case, the left lateral position was found to relieve the patient’s dyspnea, which corresponded with an increase in the tracheal lumen diameter by CT imaging. So, a left lateral decubitus position was used for the patient. Postoperatively, the patient exhibited no evidence of tracheal edema or stridor and experienced earlier extubation. These preliminary findings suggest a potential pathway for optimizing complex airway management that merits further systematic investigation.

However, this method has certain limitations, as the airway-dilating effect induced by postural changes may be substantially diminished or completely eliminated under the following pathophysiological conditions: 1) Tumor infiltration involving more than three-quarters of the airway circumference, leading to the destruction of tracheal cartilage or membranous structures (18, 27); 2) Severe fibrotic or calcified airway wall remodeling, such as post-tuberculosis or post-radiation stenosis (28); 3) Extrinsic compressive stenosis accompanied by tumor invasion of the airway wall, resulting in mechanical fixation of the lumen (20); 4) End-stage tracheomalacia with complete cartilage destruction (29); 5) Acute inflammatory edema causing luminal occupation (30). The underlying commonality among these conditions is the irreversible impairment of airway wall compliance and loss of elastic recoil capacity.

Based on biomechanical evaluations of the airway wall, we propose that the presence of full-thickness structural damage accompanied by multidirectional deterioration in mechanical properties including synchronous impairment of radial compliance, axial extensibility, and circumferential stress-bearing capacity, should be established as an objective anatomical criterion for anticipating treatment failure following postural adjustment. This criterion is applicable across all pathological conditions that compromise the structural integrity of airway wall, including but not limited to: malignant tumor infiltration, fibrotic scar stenosis, and extrinsic compressive lesions involving airway wall invasion (21). Furthermore, this case also provides insights into two key areas of airway management that require improvement in the future. First, the development of a standardized scoring system to assess stenosis severity, lesion location, and underlying pathology would facilitate improved prioritization of treatment and selection of appropriate interventions. Second, combining 3D virtual bronchoscopy with computational fluid dynamics can revolutionize surgical planning through airflow simulation, surgical rehearsal, and outcome prediction, particularly in the contexts of stent placement, resection strategy, and the prediction of complication.

## Conclusion

Giant retrosternal goiters present unique anesthetic challenges attributable to their anatomical location, thereby necessitating a multidisciplinary collaboration involving anesthesiologists, thyroid surgeons, and radiologists. Effective airway management is of utmost importance, with preoperative CT being critical for evaluating tracheal compression and displacement. Postural intervention (lateral position, head-up or semi-sitting position) may alleviate patient symptoms and optimize conditions for tracheal intubation. A dual-guidance technique combining video laryngoscopy with FOB for awake endotracheal intubation should be a feasible airway management strategy for patients with critical tracheal stenosis. Furthermore, ECMO support should be preemptively considered for critical proximal airway obstruction or severe cardiopulmonary compromise, with perfusionists on standby.

## Data Availability

The original contributions presented in the study are included in the article/**Supplementary Material**. Further inquiries can be directed to the corresponding authors.

## Supplementary material

Supplementary Figure 1 Bilateral thyroid ultrasonography identified multiple nodules. On the left side, two larger nodules measuring approximately 4.3 × 2.6 cm **(A)** and 5.6 × 4.8 cm **(B)** were observed. On the right side, a larger nodule measuring approximately 3.7 × 3.3 cm was identified **(C)**.

Supplementary Figure 2 The imaging of preoperative and postoperative lung computed tomography (CT). Postoperative lung CT imaging demonstrated a complete resolution of airway compression and stenosis compared to the preoperative condition.

Supplementary Figure 3 The imaging of preoperative and postoperative chest radiography. Postoperative chest radiograph demonstrated resolution of airway compression and restoration of tracheal midline alignment, compared with preoperative chest radiograph.
