# Supplementary figures and images for: Case Report: Awake lateral decubitus intubation for a patient with critical tracheal stenosis secondary to retrosternal goiter: salvaging a 2 mm airway without ECMO support
Source: Front Oncol. 2026 Feb 16;16:1712391. doi: 10.3389/fonc.2026.1712391 (PMC12950583; doi:10.3389/fonc.2026.1712391)

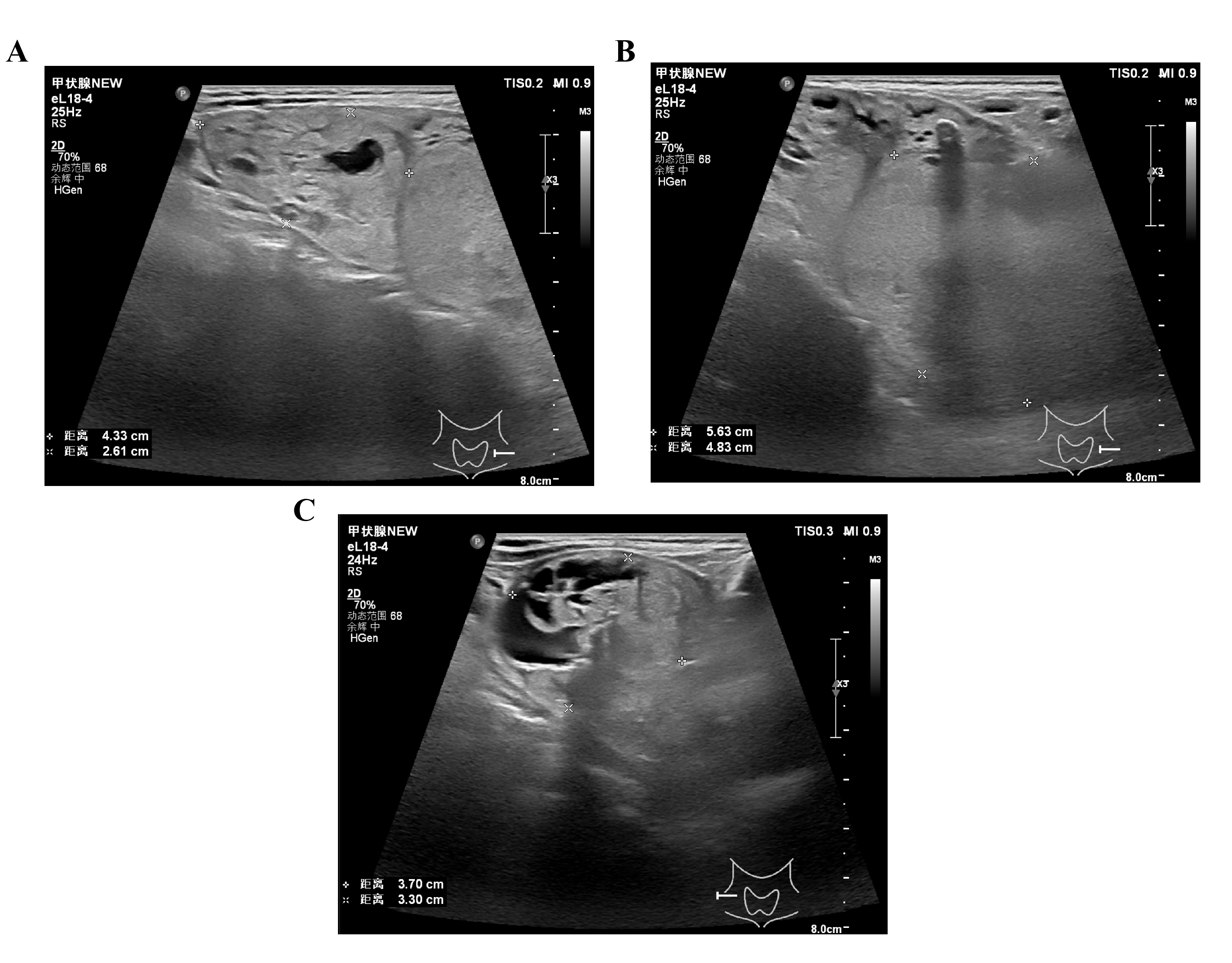

Supplement: Supplementary Figure 1 — Bilateral thyroid ultrasonography identified multiple nodules. On the left side, two larger nodules measuring approximately 4.3 × 2.6 cm (A) and 5.6 × 4.8 cm (B) were observed. On the right side, a larger nodule measuring approximately 3.7 × 3.3 cm was identified (C). [file Image1.tif]

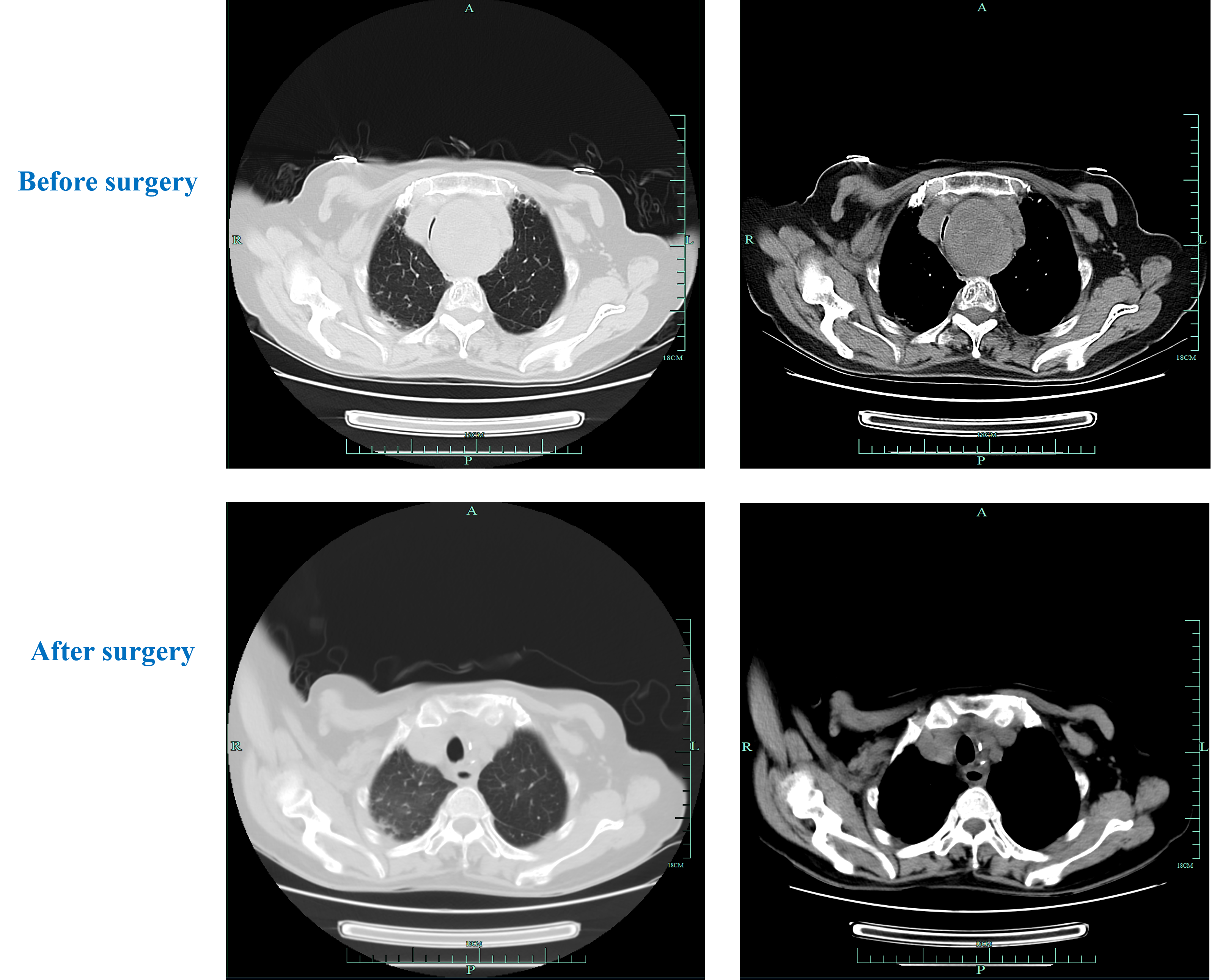

Supplement: Supplementary Figure 2 — The imaging of preoperative and postoperative lung computed tomography (CT). Postoperative lung CT imaging demonstrated a complete resolution of airway compression and stenosis compared to the preoperative condition. [file Image2.tif]

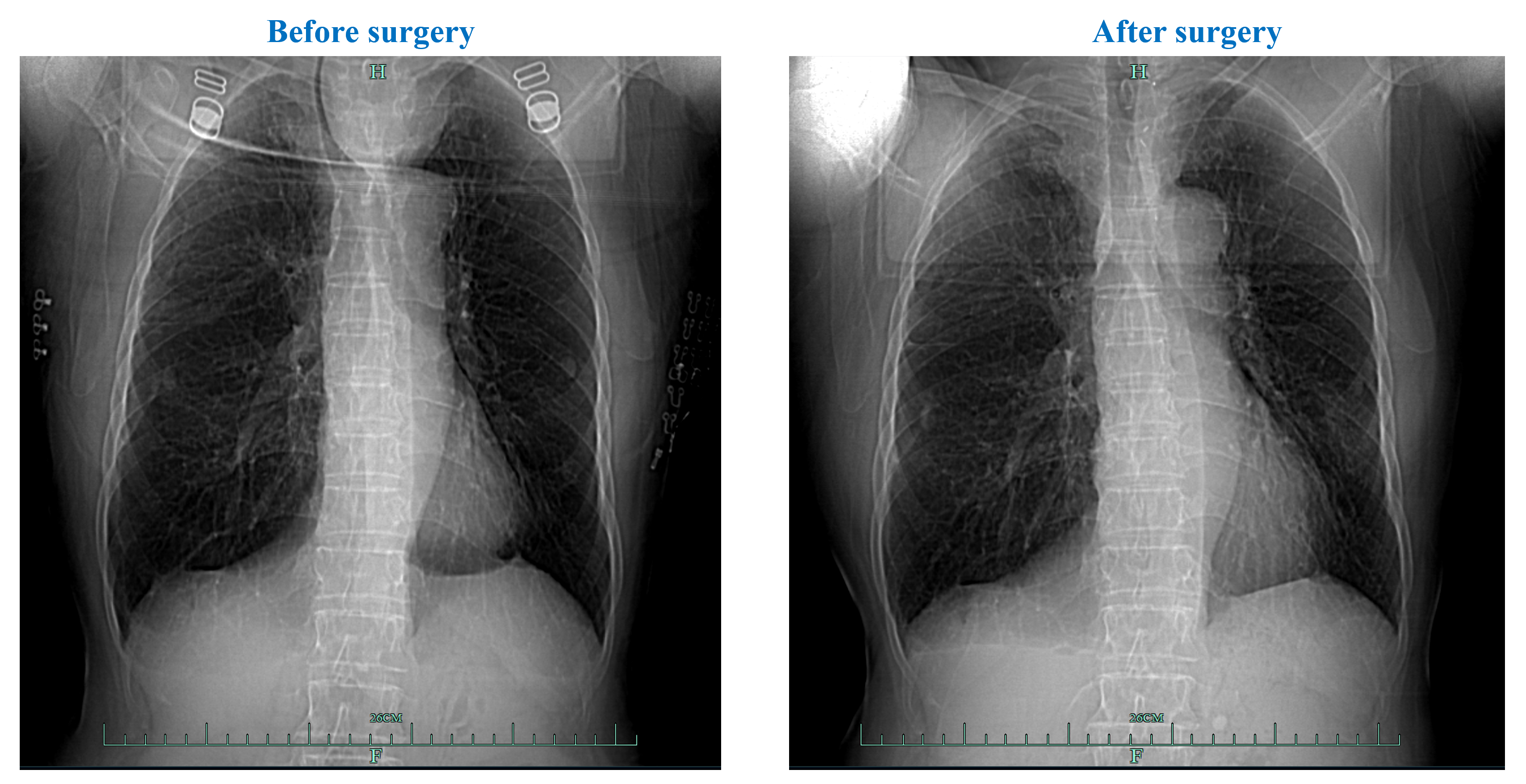

Supplement: Supplementary Figure 3 — The imaging of preoperative and postoperative chest radiography. Postoperative chest radiograph demonstrated resolution of airway compression and restoration of tracheal midline alignment, compared with preoperative chest radiograph. [file Image3.tif]
